# Mr. Warburton's Bill

**Published:** 1829-07-01

**Authors:** 


### XXXIV.

### MR. WARBURTON'S BILL.

An Act so deeply affecting the interests of medical science, deserves a permanent record in our pages; but, like all acts, it is so overloaded with words, that we shall be able to give the substance of it in a small compass.

The first clause describes the penalty for disinterring or stealing bodies on the old plan—and that is, imprisonment in the common jail or house of correction, with or without hard labour, for a term not exceeding, for the first offence, *six months*, for the second, *two years*.

The second clause appoints a commission, under the secretary of state, consisting of not fewer than seven persons, for licensing schools of anatomy, the majority of which commission shall be non-professional persons. These commissioners are to hold quarterly meetings for granting licenses—and the fees of licenses are to go to the liquidation of the expenses of the act. These commissioners are to be converted from time to time into visitors or inspectors, to inquire into the state of any school or place of dissection, and to report thereon. No commissioner or visitor shall be a teacher of anatomy or connected with a dissecting-room. These visitors shall have the power to summon before them any person connected with anatomical tuition, and examine them on oath, "touching any matters relating to the execution of this act," under a penalty of fifty pounds for non-compliance. The commissioners in question, are empowered to make a code of rules for the regulation of anatomical schools. The following is the marrow of the Act.

"And be it Enacted, That it shall be lawful for any party to whom a License shall have been granted by the said Commissioners, or to any person acting by authority of such party, so long as such license shall remain in force under such regulations as may be prescribed by the rules and orders of the said Commissioners, to receive from or by order of any of the persons hereinafter authorized to deliver up the same, any such human body as is hereinafter permitted to be delivered up, and to remove such body from the place of delivery to the place appointed for dissection, in the license to such party granted, and there to dissect the same.

And be it further Enacted, That when any person shall die during imprisonment in any prison, or shall die in any hospital or workhouse, and the body of such person shall not be claimed as hereinafter mentioned, or the disposition of such body shall not be otherwise provided for by law, it shall be lawful for the party having the custody of the person so dying in prison as aforesaid, and for the party having the care of the person dying in any hospital or workhouse as aforesaid, to deliver up the body of such person to any party duly licensed under this Act, or to the authorized agent of such party: Provided always, That if within *Seventy-two* hours after the death of any such person as aforesaid, any person shall attend to remove the body, and there shall be sufficient reason to believe that such body if delivered to such person, will be by him duly buried and not delivered up for dissection, the same shall be delivered up to the person so attending as aforesaid: And provided also, That if within the said period of *Seventy-two* hours, any person representing himself to be a relative of the deceased, shall request that such body may not be delivered up to be dissected, and there is sufficient reason to believe that the person making such request is really a relative of the deceased, and no nearer relative has made any request to the contrary, such body shall not be delivered up for dissection.

"Provided always, and be it further Enacted, That nothing herein contained shall prevent the due holding of inquests by the coroners, but that in all cases where a coroner's inquest may be necessary, no body shall be delivered up by virtue of this Act, until such inquest has been held.

"And be it Enacted, That if any person shall, during his life time, by any instrument in writing, attested by two or more witnesses, declare that he is desirous that his body after death may be delivered up for dissection, it shall be lawful for the executors, administrators or next of kin of every such person, to deliver up, if they shall think fit, the body of such person for dissection; provided that *Three* days previous to such delivery, they shall have given notice to the overseers of the parish in which such person died, of their intention so to deliver up the body, and shall have sent to such overseers, together with such notice, a copy of the instrument, declaratory of the desire of the deceased, and a certificate signed by *Three* or more physicians, surgeons or apothecaries, that the deceased came fairly by his death.

"And be it Enacted, That if in any case not hereinbefore provided, any party shall be desirous of delivering up, and any party of receiving, any human body for dissection, or if any party, at any other place than a licensed Dissecting School, shall be desirous of dissecting, it shall be lawful for the said Commissioners, if they shall think fit, to grant a special License for any such purpose as aforesaid, and to charge for such License any sum not exceeding *Two pounds*.

"And be it enacted, That every party receiving any human body for dissection, shall demand and receive, together with the body, a Certificate, stating at what hour, on what day, in what month and in what year, by whom or by whose authority, and to whom or on whose account the body was delivered up, the date and place of death, the sex and (as far as it is known at the time) the christian and surname, parent's age, trade or occupation, and last place of abode of such person; and the party delivering up the body shall deliver and sign such certificate, and the party receiving the body shall enter, or cause to be entered, a copy of every such certificate in a book to be kept by him for that purpose; and every party licensed under this Act shall produce such book, or a copy thereof, or extract therefrom, whenever required to do so by the said Commissioners.

"And be it Enacted, That every party licensed under this Act, shall, after dissection, at his own cost, enclose the remains of every body by him or by his authority dissected, in a separate coffin, and shall at his own cost within *Twenty-one* days after the receipt of such body, decently bury or cause to be buried the remains of the same, with the rites of christian burial, or with such other funeral rites and solemnities as accord with the religious creed of the deceased, or are customary in that part of the kingdom where such burial shall take place, and shall cause entry to be made in the parish register of the parish where such burial shall take place, of the name, age and abode of the person buried, and of the date of the burial, and of the name of the minister officiating thereat.

"And be it Enacted, That if at any time the said Commissioners shall recommend to His Majesty's Principal Secretary of State for the Home Department for the time being, that any License granted by them to any party by virtue of this Act, should, before the expiration of such License be suspended for a time or revoked altogether, or upon its expiration should not be renewed, it shall be lawful for such Secretary of State, by an instrument under his hand and seal to be transmitted to such party, to suspend for a time or to revoke altogether such License, or to prohibit such party from having the same renewed: Provided always, That a written notice of such recommendation shall be sent by the clerk of the said Commissioners to such party *Fourteen* days at least before the transmission thereof to such Secretary of State.

And be it Enacted, That if any person after the *First day of October* in the present year, shall keep a School for the dissection of human bodies, or shall knowingly permit dissection to be taught or practised in any place to him belonging, without having obtained a License for that purpose, in the manner directed by this Act, every person so offending shall forfeit a sum not exceeding *One hundred pounds*.

And be it Enacted, That if any person shall, after the day last mentioned, knowingly receive, remove, deposit or possess any human body, with a view to dissection, without a License from the said Commissioners, or without the authority of a person having such a License, every person so offending shall forfeit a sum not exceeding *Fifty pounds*.

"And be it Enacted, That if any person shall, after the day last mentioned, dissect a human body at any place not set forth in any License in force, and by the said Commissioners granted, every person so offending shall forfeit a sum not exceeding *Fifty pounds* ; provided, that nothing herein contained shall be understood to extend to an examination post mortem of any human body required to be made by legal authority or permitted to be made by the relatives of any deceased person, or in any hospital or hospitals, such examination being made at the place where the person died.

"And be it Enacted, That if any party licensed by the said Commissioners, or any person acting by authority of such party, shall, after the day last mentioned, knowingly receive, remove or deliver up any human body at any other time than shall be specified in the rules and orders of the said Commissioners, or shall receive any human body from any person not authorized by virtue of this Act to deliver human bodies up, or shall remove or deliver any human body to or receive any human body at any other place than that set forth in the License to such party granted, or shall on any occasion wantonly expose any human subject to public view, or shall receive any human body without such a certificate as aforesaid, every such offender shall forfeit a sum not exceeding *Fifty pounds*.

"And be it Enacted, That if any party licensed by the said Commissioners shall, after the day last mentioned, on receiving any human body, neglect to register the certificate by him hereinbefore directed to be taken, or shall wilfully make any false entry in such register, or shall neglect or refuse to produce to the said Commissioners at their desire the book of registry hereinbefore directed to be kept by such party, or a copy thereof or extract therefrom, or shall, on burying the remains of any such body, wilfully neglect to register such burial in the register of the parish where such burial shall take place in the manner hereinbefore directed, or shall wilfully make or cause to be made any false entry of such burial in any parish register, every party so offending shall forfeit a sum not exceeding *Fifty pounds*.

"And be it Enacted, That if any party licensed by the said Commissioners shall, after the day last mentioned, omit to bury in the manner hereinbefore directed the remains of any human body by him or by his authority received for dissection or dissected, every such offender shall forfeit a sum not exceeding *Fifty pounds*.

"And be it Enacted, That from and after the day last mentioned, if any person having authority by virtue of this Act to deliver up human bodies for dissection, shall deliver such up to any party not licensed to receive bodies for dissection, every such person shall forfeit a sum not exceeding *Twenty pounds*.

"And be it enacted, That if any person duly summoned to attend as a witness in any proceeding under this Act shall wilfully and without sufficient cause neglect or refuse to attend, or if he shall attend and shall refuse to be sworn to give evidence, every such person shall for every such offence forfeit a sum not exceeding *Fifty pounds*.

"And be it Enacted, That it shall and may be lawful for the said Commissioners, in the name of their clerk, or for any other person or persons, to sue for the penalties and forfeitures granted by this Act.

"And be it Enacted, That all monies to be received for any Licenses to be granted by virtue of this Act, shall be retained by the clerk to the said Commissioners, and from such monies any expenses that may be incurred in the execution of this Act shall upon the order of the said Commissioners to be paid, and such clerk of the Commissioners shall keep a true account of all such receipts and disbursements, and shall at all times exhibit such account to the said Commissioners when required by them so to do, and shall make up such account to the *Thirtieth* day of *June* in each year, and such yearly account, when examined and approved by the said Commissioners, shall be signed by *Three* or more of them, and shall then be transmitted by such clerk to the Lords Commissioners of His Majesty's Treasury, who shall thereupon, if they shall think fit, direct the balance, if any, that may be in the hands of such clerk to be paid into the Exchequer to the account of the Consolidated Fund of the United Kingdom of Great Britain and Ireland; *but if there shall be a balance due to such clerk it shall be lawful for the Lords Commissioners of His Majesty's Treasury, or Three or more of them, to cause the requisite sum to be issued and paid from time to time out of any money in the Exchequer applicable to the growing produce of the Consolidated Fund.*"

The remainder of the Act relates to the recovery of penalties, and is quite of a legal or technical nature, devoid of interest.

We have understood that the Act, as above sketched out, does not give satisfaction to those connected with anatomical tuition. With the exception of one or two clauses, we really see but little ground for dissatisfaction. The great difficulty will be in the Christian burial vouchsafed for the disjecta membra of the dissected bodies. It will be almost as puzzling an operation to preserve the identity of individuals, on sepulture after dissection, as at the final resurrection! Each identification will require the interposition of a miracle! That this clause is a placebo for popular prejudice there can be no doubt—and that it will give origin to much farcical ceremony, more beneficial to the stomachs of the undertaker, Parson, and grave-digger, than to the souls of the dead, there can be as little doubt among all thinking creatures. Its worst effect will be the tax on the student. No doubt, contractors will be found to undertake this ceremony at a small expense.

That some difficulties will be thrown in the way of the beneficial operation of this Act, by the "brief authorities," namely, the parish officers, church-wardens, overseers, et hoc genus omne, is to be reasonably expected. But still we shall have great reason to be grateful to the legislature—and more especially to Mr. Warburton and Mr. Peel—for an enactment which opens the door to almost every facility that can be desired for the prosecution of anatomical study.

